# Study of a hysteresis window of FinFET and fully-depleted silicon-on-insulator (FDSOI) MOSFET with ferroelectric capacitor

**DOI:** 10.1186/s40580-020-00230-x

**Published:** 2020-06-01

**Authors:** Chankeun Yoon, Seungjun Moon, Changhwan Shin

**Affiliations:** Department of Electrical and Computer Engineering, Sungkyunkwan University, Suwon, 16419 Korea

**Keywords:** Hysteresis, Fully-depleted silicon-on-insulator (FDSOI) device, Fin-shaped field-effect-transistor (FinFET), Ferroelectric capacitor

## Abstract

In this work, the measured electrical characteristics of a fully depleted silicon-on-insulator (FDSOI) device and fin-shaped field-effect transistor (FinFET), whose gate electrode is connected in series to the bottom electrode of a ferroelectric capacitor (FE-FDSOI/FE-FinFET), are experimentally studied. The hysteretic property in input transfer characteristic of those devices is desirable for memory device applications, so that the understanding and modulating the hysteresis window is a key knob in designing the devices. It is experimentally observed that the hysteresis window of FE-FDSOI/FE-FinFET is decreased with (i) increasing the area of the ferroelectric capacitor and/or (ii) decreasing the gate area of baseline FET. The way how to control the hysteresis window of FE-FDSOI/FE-FinFET is proposed and discussed in detail.

## Introduction

Digital computer has been faced with a few technical issues/limits, primarily because of ever-increasing power density [1, 2]. To address the bottleneck, neuromorphic computation system, which can achieve (i) parallel networks and (ii) energy-efficient and robust computation by mimicking the biological brain, has received lots of attentions [3, 4]. The neuromorphic networks consist of neuron circuits and synaptic devices. Each neuron circuit is connected with hundreds of synaptic devices, and therefore, the development of synaptic devices that can implement high density is of importance [5]. The synaptic devices in neuromorphic system have been widely studied with non-volatile memories (i.e., phase-change memory (PCM) [6], resistive change memory (RRAM) [7], conductive bridge type memory (CBRAM) [8], and ferroelectric field-effect transistor (FeFET) [9]). Among them, FeFET would be a prominent candidate for the synaptic device because of its compatibility to complementary metal–oxide–semiconductor (CMOS) fabrication process [9]. Besides, FeFET can be designed as a low power logic device, if a negative capacitance effect in ferroelectric materials is used [10–12]. Ferroelectric material maintains its polarization state even in the absence of an external electric field. Due to its unique property, the hysteretic properties and steep-switching behaviour without hysteresis in the input transfer characteristic of FeFET are reported. The hysteretic properties can be used in designing FeFET as a non-volatile memory as well as artificial synaptic device. On the other hand, the hysteresis-free steep switching characteristic can be used in designing FeFET as a low power logic device [13]. Therefore, understanding/modulating the hysteresis window as well as providing the device design guideline to modulate the hysteresis window should be investigated.

There are two primary pathways for fabricating FeFETs: (i) inserting a ferroelectric layer within the gate stack of baseline FET, and (ii) connecting a ferroelectric capacitor in series to the top gate electrode of baseline FET. The latter gives opportunities to test various combinations (i.e., ferroelectric capacitor + baseline FET), and thereby, modulating the hysteresis window of FeFET [14]. In this work, the impacts of (i) top-electrode area of ferroelectric capacitor and (ii) device dimensions of baseline FET on the hysteresis window are experimentally and systematically investigated. As one of the cutting-edge transistor structures in industry, fully-depleted silicon-on-insulator (FDSOI) device and fin-shaped FET (FinFET) are chosen and fabricated as the baseline FET in this work. The experimental results indicate that the hysteresis window of FeFET can be modulated by changing the top-electrode area of ferroelectric capacitor as well as the device dimensions of baseline FET, i.e., as the top-electrode area of ferroelectric capacitor is increased (and/or the device dimensions of baseline FET are decreased), the hysteresis window of FeFET is decreased.

## Fabrication and measurement

**Fig. 1:**
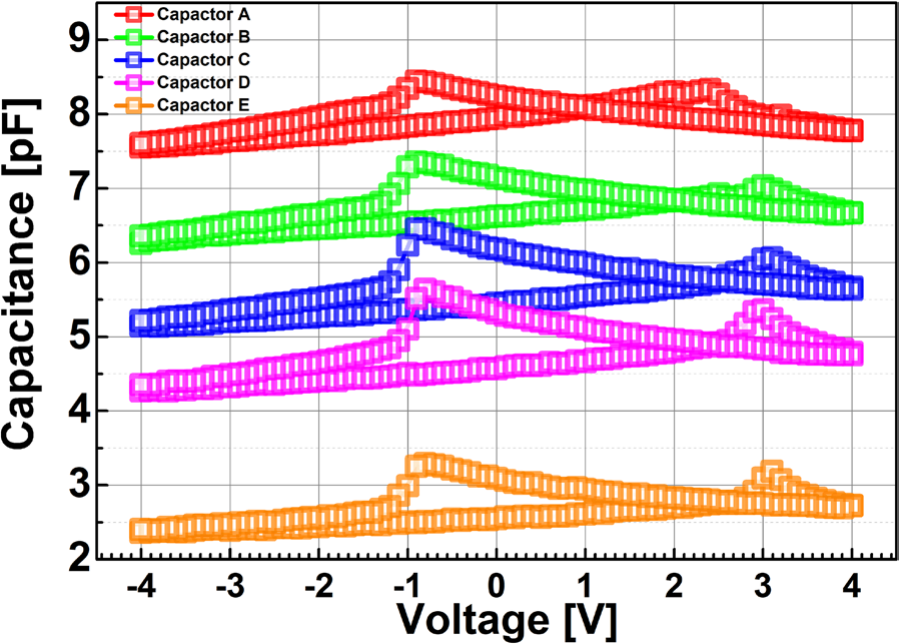
Measured capacitance vs. voltage applied to the ferroelectric capacitor with a few areas. The area of ferroelectric capacitor A, B, C, D, and E is 50 × 50 μm^2^, 45 × 45 μm^2^, 40 × 40 μm^2^, 30 × 30 μm^2^, and 20 × 20 μm^2^, respectively

In order to fabricate a ferroelectric capacitor, a 20-nm-thick La_0.7_Sr_0.3_MnO_3_ (LSMO) bottom electrode was deposited on an NdScO_3_ (NSO) substrate. Afterwards, a 60-nm-thick Pb(Zr_0.2_Ti_0.8_)O_3_ (PZT) layer was formed using the pulsed laser deposition (PLD) technique. Finally, a 60-nm-thick Au/Ti/Au top electrode was deposited with various areas. Figure 1 shows the measured capacitance-versus-voltage of ferroelectric capacitors with a few different areas, indicating that the capacitor with a larger top-electrode area has a higher capacitance.

The main features of fully-depleted silicon-on-insulator (FDSOI) device were as follows: the gate stack was made of 2.5-nm-thick SiON/TiN. The thickness of SOI layer and buried oxide (BOX) are 20 nm and 10 nm, respectively. The main features of FinFET are as follows: the fin height, the number of fins, gate to source/drain length, and equivalent oxide thickness (EOT) are 40 nm, 5, 90 nm and 1.4 nm, respectively.

**Fig. 2:**
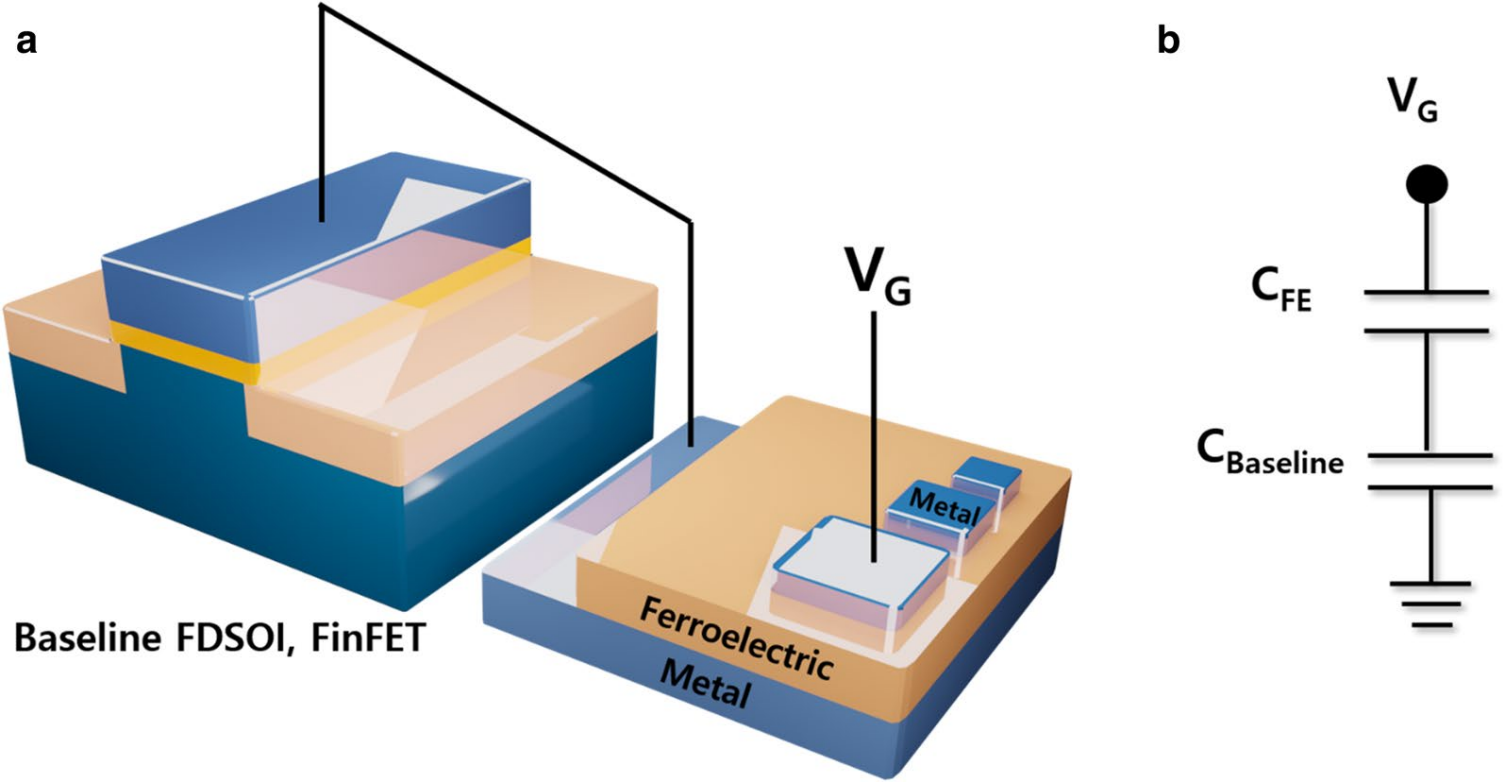
**a** Conceptual view showing the ferroelectric capacitor connected in series to the gate electrode of baseline FET. Note that the ferroelectric capacitor is connected in series to FDSOI device or FinFET in this work. **b** Capacitive circuit schematic of ferroelectric capacitor connected in series to the FDSOI device (FE-FDSOI) or FinFET (FE-FinFET)

In order to modulate the hysteresis window, various combinations between baseline devices and ferroelectric capacitors are necessary, so that the bottom electrode of ferroelectric capacitor was connected in series to the top gate electrode of baseline FET (see Fig. 2a). The capacitive circuit schematic of the series-connected device (denoted as FE-FDSOI or FE-FinFET) is drawn in Fig. 2b. It is noteworthy that there exists two main capacitive components in FE-FDSOI and FE-FinFET, i.e., the ferroelectric capacitance ($C_{FE}$) and the capacitance of baseline FET ($C_{Baseline}$). By applying a gate voltage to the top-electrode of ferroelectric capacitor in FE-FDSOI/FE-FinFET, the electrical characteristics of them were measured, and then they were compared with the electrical characteristics of baseline FDSOI device and FinFET. Note that the drain current (I_DS_) versus gate voltage (V_GS_), and capacitance (C) versus voltage (V) were measured using the Keithley 4200A-SCS analyzer at room temperature (300 K).

## Results and Discussion

### Impact of modulating the top-electrode area of ferroelectric capacitor

**Fig. 3:**
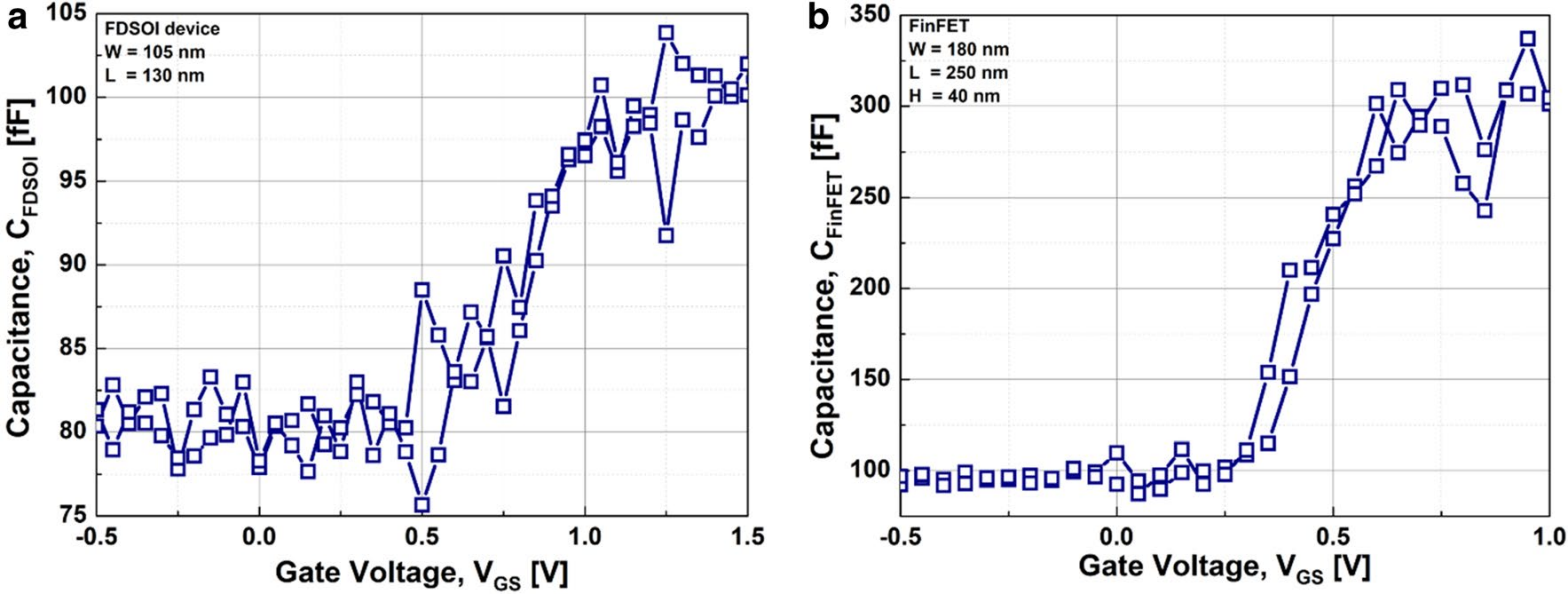
Measured capacitance vs. gate voltage of baseline **a** FDSOI device, **b** FinFET

**Fig. 4:**
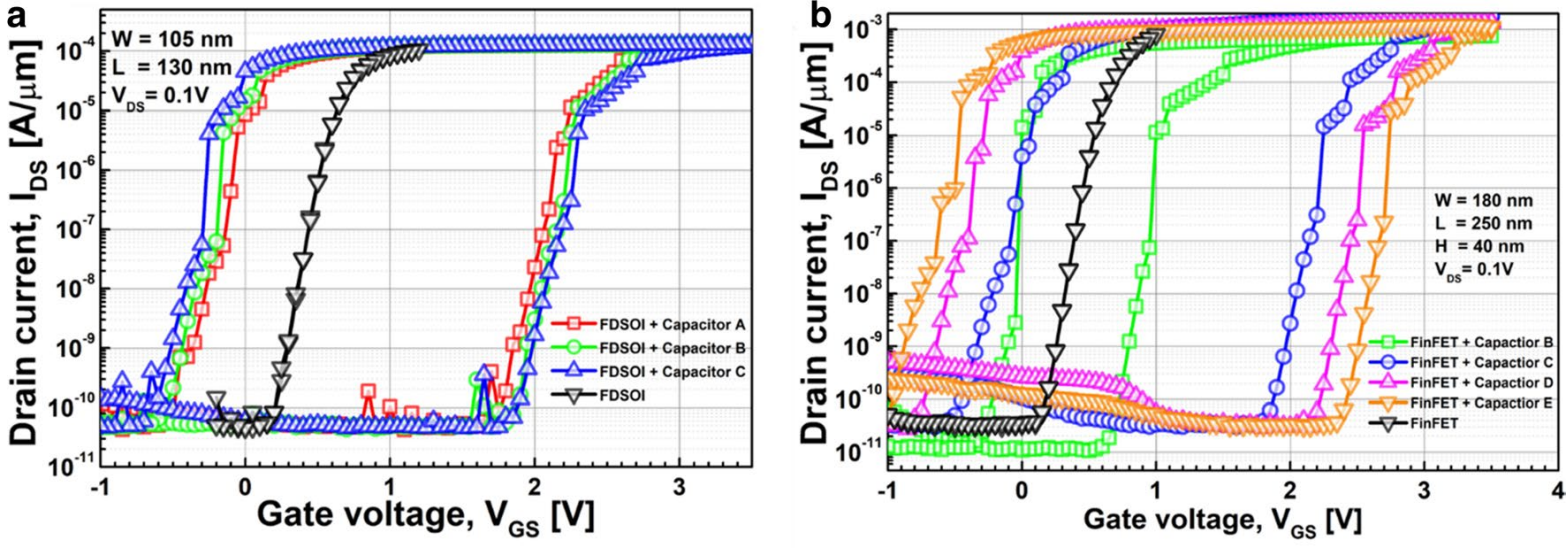
Measured drain current vs. gate voltage of **a** FE-FDSOI, **b** FE-FinFET with various areas of ferroelectric capacitor

**Fig. 5:**
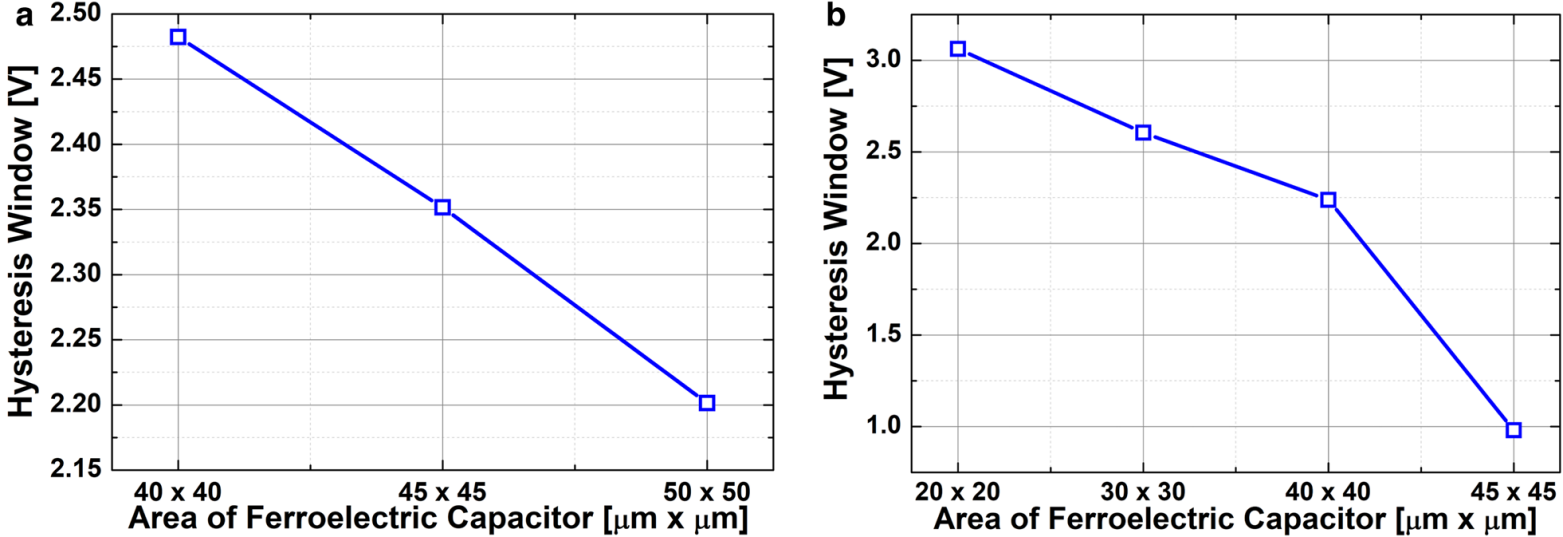
Measured hysteresis window of **a** FE-FDSOI, **b** FE-FinFET vs. the area of ferroelectric capacitor

Figure 3 shows the measured capacitance-vs.-voltage of baseline FDSOI device (C_FDSOI_) and FinFET (C_FinFET_). It is noteworthy that C_FDSOI_ and C_FinFET_ are not identical to each other, because the FDSOI device and FinFET have its own device structure and dimension. Figure 4 shows the measured input transfer characteristics of FE-FDSOI/FE-FinFET at V_DS_ of 0.1 V. Note that the measured input transfer characteristics of stand-alone FDSOI and FinFET are included in Fig. 4, as well. The gate voltage sweep range of FE-FDSOI/FE-FinFET was from − 4 V to + 4 V and − 3.5 V to + 3.5 V, respectively. The reverse gate voltage sweep was conducted after the forward sweep with identical voltage range. The drain current of FE-FDSOI was normalized to the channel width of baseline FDSOI device. And, the drain current of FE-FinFET was normalized to the effective channel width (i.e., fin width + 2 × fin height) of baseline FinFET device. As shown in Fig. 4, the hysteretic properties were clearly observed in the measured input transfer curves of FE-FDSOI and FE-FinFET. Herein, it turned out that the hysteresis window of FE-FDSOI and FE-FinFET can be controlled by modulating the top-electrode area of ferroelectric capacitors (while maintaining the dimension parameters of baseline FDSOI and FinFET such as channel length and width). Figure 5 indicates that the hysteresis window of FE-FDSOI and FE-FinFET is decreased with increasing the top-electrode area of ferroelectric capacitor. In this work, the hysteresis window of FE-FDSOI and FE-FinFET was measured in terms of the resultant difference in the threshold voltages (V_th_) during the forward and reverse sweeps, as follows:

1 $$Hysteresis = V_{th\_FE - FDSOI/FinFET\_forward} - V_{th\_FE - FDSOI/FinFET\_reverse}$$

The voltage across the ferroelectric capacitor during the forward sweep can be estimated as the difference in threshold voltages of the FE-FDSOI/FE-FinFET and baseline FDSOI/FinFET during forward sweep, as follows:

2 $$V_{Ferroelectric\_forward} = V_{th\_FE - FDSOI/FinFET\_forward} - V_{th\_FDSOI/FinFET\_forward}$$

Likewise, the voltage across the ferroelectric capacitor during the reverse sweep can be estimated as the difference in threshold voltages of the FE-FDSOI/FE-FinFET and baseline FDSOI/FinFET during reverse sweep, as follows:

3 $$V_{Ferroelectric\_reverse} = V_{th\_FE - FDSOI/FinFET\_reverse} - V_{th\_FDSOI/FinFET\_reverse}$$

By combining the Eqs. (2) and (3) into the Eq. (1), the hysteresis of FE-FDSOI/FE-FinFET is given, as follows:

4 $$Hysteresis = V_{Ferroelectric\_forward} - V_{Ferrelectric\_reverse}$$

**Fig. 6:**
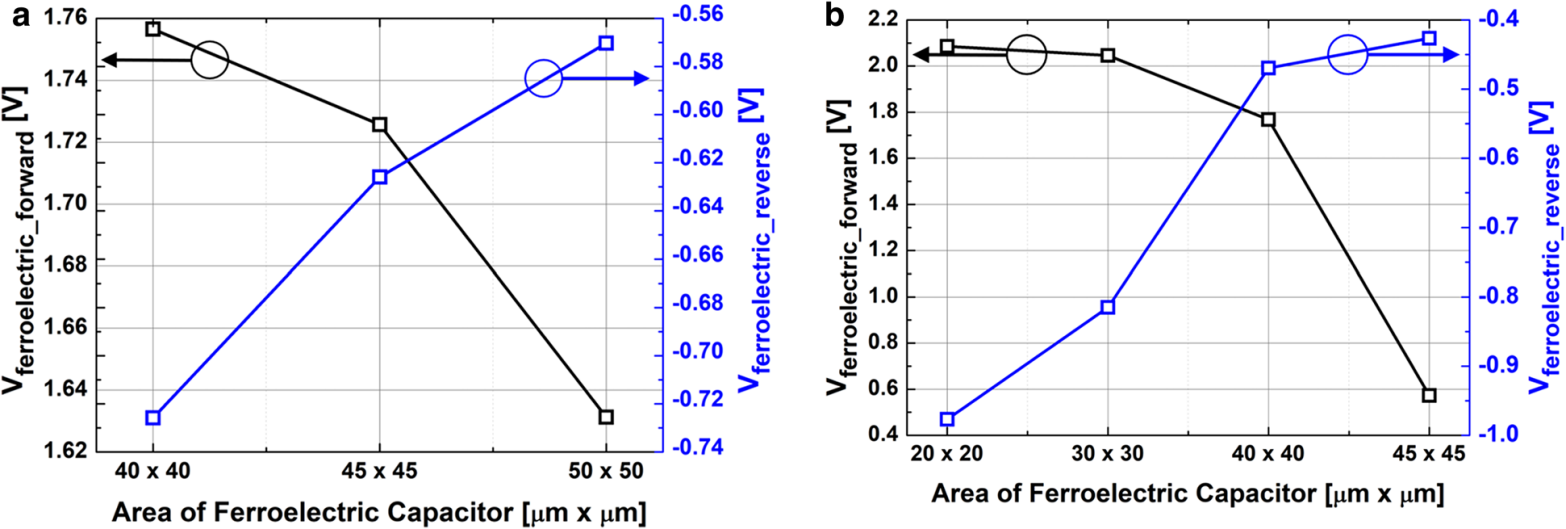
Measured voltage across the ferroelectric capacitor during forward sweep and reverse sweep vs. the area of ferroelectric capacitor: **a** FE-FDSOI, **b** FE-FinFET

According to the voltage divider rule, the voltage across the ferroelectric capacitor (V_FE_) can be expressed as follows:

5 $$V_{FE} = \frac{{C_{baseline} }}{{C_{FE} + C_{baseline} }}V_{G} ,$$

where C_FE_ is the capacitance of ferroelectric capacitor, and C_baseline_ is the capacitance of baseline FDSOI/FinFET. The equation above indicates that V_FE_ is determined by (i) the capacitance of baseline FDSOI/FinFET (C_baseline_), (ii) the capacitance of ferroelectric capacitor (C_FE_) and (iii) the voltage applied to the FE-FDSOI and FE-FinFET (V_G_). As illustrated in Figs. 1 and 3, C_FE_ is much larger than C_baseline_. Therefore, in this work, C_FE_ affects V_FE_ much more than C_baseline_. Meanwhile, C_FE_ is proportionally increased with increasing its top-electrode area (see Fig. 1). This enables to decrease V_FE_ in the Eq. (5). Figure 6 shows that the voltage across ferroelectric capacitor during the forward sweep is decreased as the top-electrode area of ferroelectric capacitor is increased. Likewise, the absolute value of V_FE_ during reverse sweep is decreased as the area is increased. Conforming to the definition in Eq. (4), the hysteresis window of FE-FDSOI/FE-FinFET is decreased as the top-electrode area of ferroelectric capacitor is increased, because of a smaller voltage drop across the ferroelectric capacitor with a larger top-electrode area. Meanwhile, it was observed that the hysteresis window is decreased as the top-electrode area of ferroelectric capacitor is increased, even at high drain voltage (not shown here). Table 1 summarizes the area of ferroelectric capacitor vs. hysteresis window. It is noteworthy that the variation of hysteresis window in FinFET is larger than that of FDSOI device, when the top-electrode area of ferroelectric capacitor changes from 45 μm × 45 μm to 40 μm × 40 μm. As mentioned above, (i) C_baseline_ of FDSOI device and FinFET and (ii) gate voltage applied to the FE-FDSOI and FE-FinFET (V_G_) are not identical to each other. Thus, (i) different C_baseline_ of FDSOI device and FinFET and (ii) V_G_ simultaneously affect V_FE_ in the FE-FDSOI and FE-FinFET. Therefore, it is rather tricky to quantitatively analyze why the hysteresis variation of FinFET was larger than that of FDSOI device when the top-electrode area of ferroelectric capacitor was changed equally in this work.

**Table 1:** The area of ferroelectric capacitor vs. hysteresis of FE-FDSOI/FE-FinFET

Area of ferroelectric capacitor (μm × μm)	Forward V_Ferroelectric_ (V)	Reverse V_Ferroelectric_ (V)	Hysteresis (V)
FE-FDSOI			
50 × 50	1.63	− 0.57	2.20
45 × 45	1.73	− 0.63	2.36
40 × 40	1.76	− 0.73	2.49
FE-FinFET			
45 × 45	0.57	− 0.43	1
40 × 40	1.77	− 0.47	2.24
30 × 30	2.05	− 0.82	2.87
20 × 20	2.09	− 0.98	3.07

### Impact of modulating the device dimensions of baseline FDSOI/FinFET devices

**Fig. 7:**
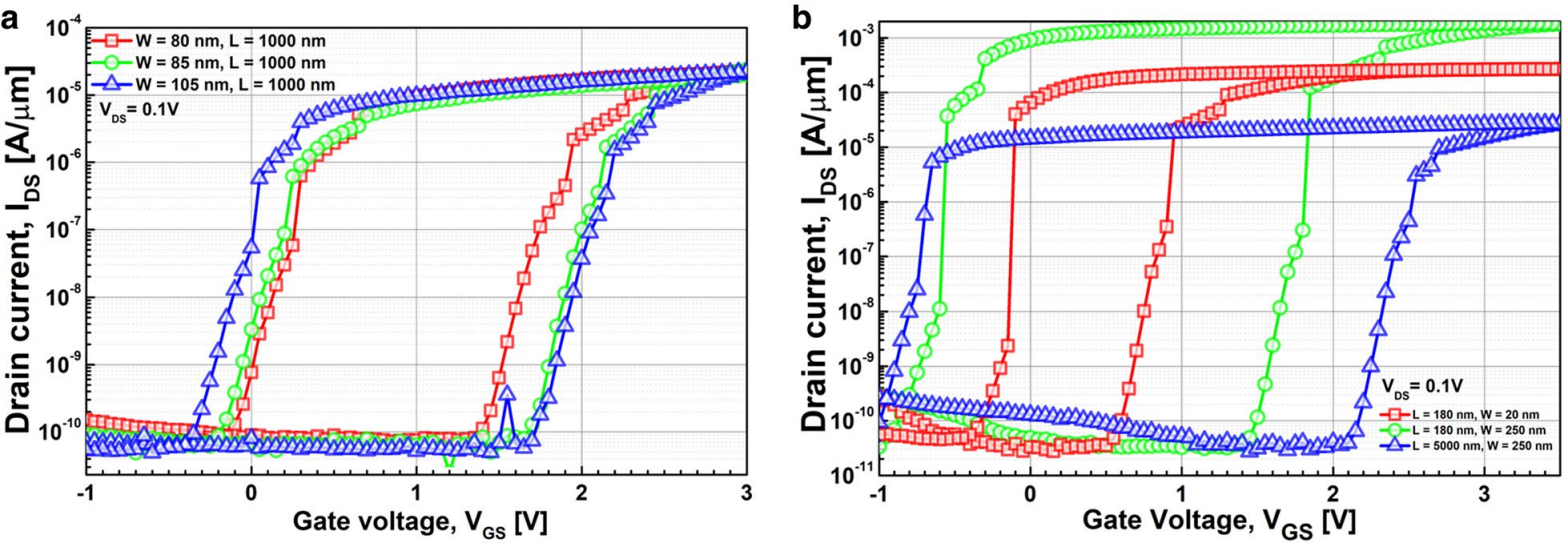
Measured drain current vs. gate voltage of **a** FE-FDSOI, **b** FE-FinFET, for various device dimensions of baseline FET

**Fig. 8:**
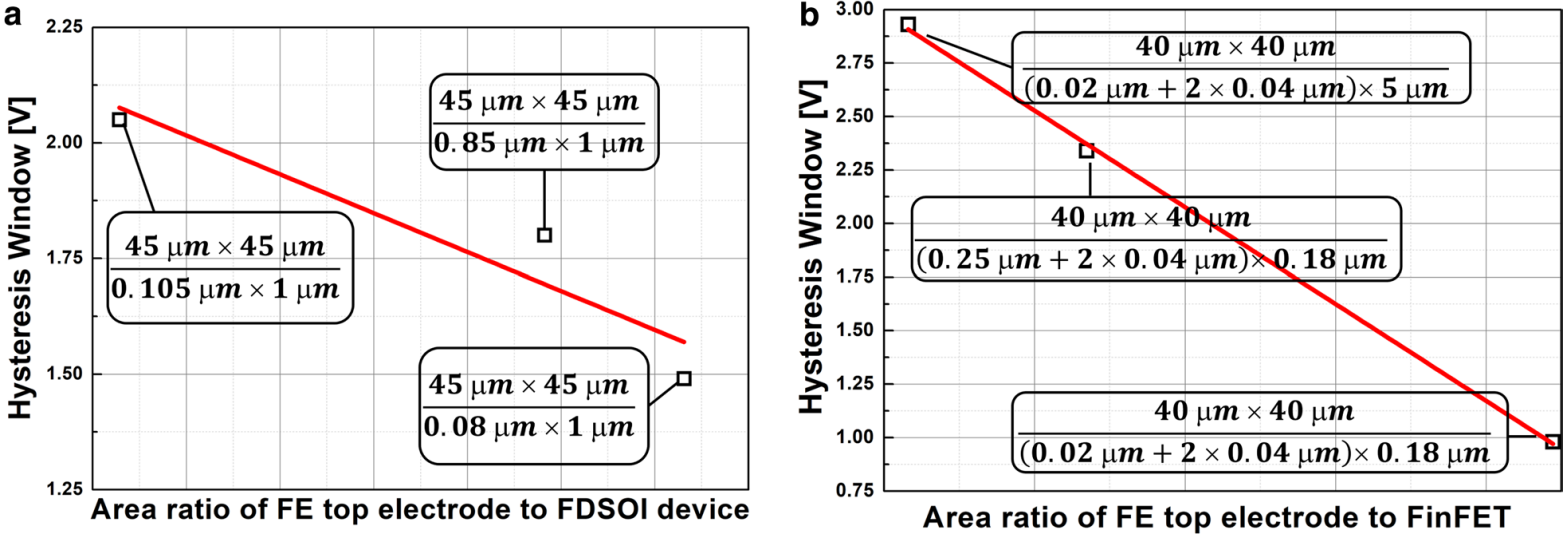
Measured hysteresis window of **a** FE-FDSOI, **b** FE-FinFET vs. the area ratio of ferroelectric capacitor top electrode to baseline FET

Figure 7 shows the measured input transfer characteristics of FE-FDSOI and FE-FinFET. The gate voltage sweep range of FE-FDSOI/FE-FinFET was from − 5 V to + 5 V and − 3.5 V to + 3.5 V, respectively. Herein, the hysteresis window of the FE-FDSOI/FE-FinFET was controlled by modulating the device dimensions of baseline FDSOI/FinFET (while maintaining the top-electrode area of ferroelectric capacitor). Specifically, the FE-FDSOI/FE-FinFET used the ferroelectric capacitor having its top electrode area of 45 μm × 45 μm and 40 μm × 40 μm, respectively. It is noteworthy that step-like behaviors in the measured input transfer characteristics were observed. These behaviors should be originated from non-ideal effects (occurred in ferroelectric materials) such as (i) leakage path in the ferroelectric material and (ii) multi-domain properties of the ferroelectric material [15, 16]. Therefore, those step-like behaviors should be addressed by fabricating a high-quality (i.e., low leakage) single domain ferroelectric. Figure 8 indicates that the hysteresis window of FE-FDSOI/FE-FinFET is decreased as the area ratio of ferroelectric capacitor top-electrode to baseline FET is increased. Herein, effective gate area of FDSOI device and FinFET was calculated as follows:

6 $${\text{Effective gate area of FDSOI device }} = {\text{ channel width}} \times {\text{channel length }}({\text{i}}.{\text{e}}.,{\text{ W}} \times {\text{L}})$$

7 $${\text{Effective gate area of FinFET }} = \, ({\text{fin width }} + { 2} \times {\text{fin height}}) \times {\text{fin length }}({\text{i}}.{\text{e}}., \, ({\text{W }} + { 2} \times {\text{H}}) \times {\text{L}})$$

**Fig. 9:**
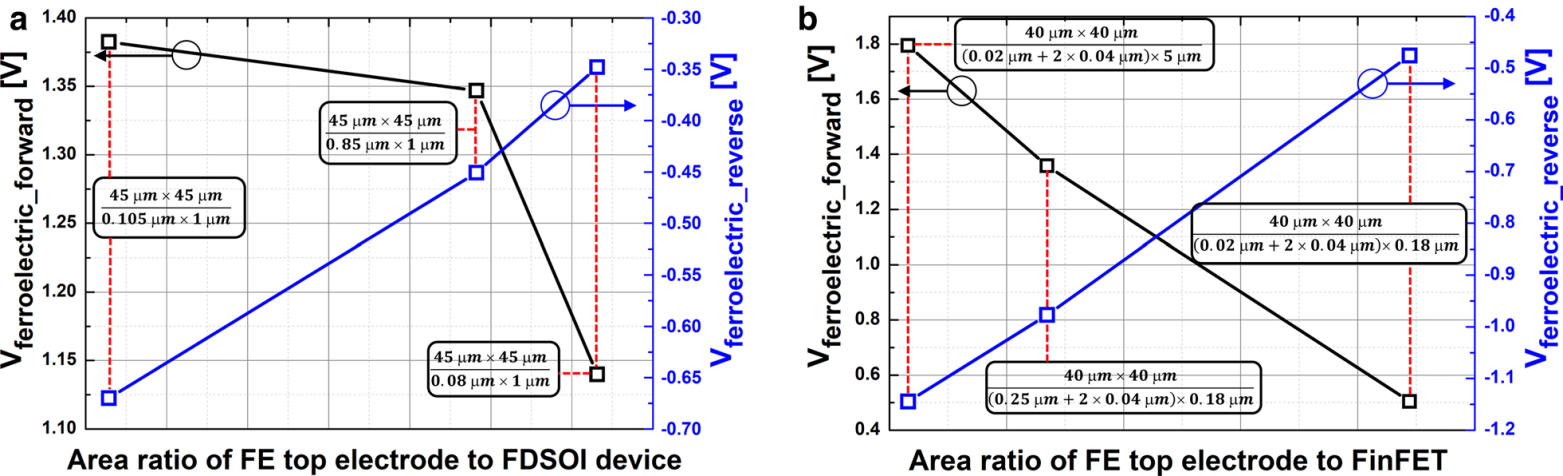
Measured voltage across the ferroelectric capacitor during forward sweep and reverse sweep vs. the area ratio of ferroelectric capacitor top electrode to **a** FDSOI device, **b** FinFET

For given top-electrode area of ferroelectric capacitor, C_FE_ remained constant. However, the capacitance of baseline FET (C_baseline_) is proportionally decreased with decreasing the device dimensions. This enables to cause V_FE_ in Eq. (5) to decrease. Figure 9 shows that V_FE_ during the forward sweep is decreased, as the area ratio of ferroelectric capacitor’s top-electrode to baseline FET is increased. Likewise, the absolute value of V_FE_ during reverse sweep is decreased as the area ratio of ferroelectric capacitor’s top-electrode to baseline FET is increased. Conforming to the definition in Eq. (4), the hysteresis window of FE-FDSOI/FE-FinFET is decreased as the area ratio of ferroelectric capacitor’s top-electrode to baseline FET is increased, because of a smaller voltage drop across the ferroelectric capacitor. Table 2 summarizes the device dimensions of baseline FDSOI/FinFET vs. hysteresis window.

**Table 2:** The device dimensions of baseline FDSOI device/FinFET vs. the hysteresis of FE-FDSOI/FE-FinFET

Gate length × width (nm × nm)	Forward V_Ferroelectric_ (V)	Reverse V_Ferroelectric_ (V)	Hysteresis (V)
FE-FDSOI			
1000 × 80	1.14	− 0.35	1.49
1000 × 85	1.35	− 0.45	1.80
1000 × 105	1.38	− 0.67	2.05
FE-FinFET			
180 × 20	0.50	− 0.48	0.98
180 × 250	1.36	− 0.98	2.34
5000 × 20	1.79	− 1.14	2.93

## Conclusion

In order to systematically modulate the hysteresis window of FE-FDSOI/FE-FinFET devices, the top-gate electrode of baseline FET was connected in series to the bottom electrode of the ferroelectric capacitor. The metal/ferroelectric/metal/dielectric/semiconductor (i.e., MFMIS) (herein, ferroelectric capacitor + FDSOI/FinFET) configuration may dilute the benefit of CMOS fabrication process compatibility due to its large device size (vs. MFIS). However, the area penalty in MFMIS structure can be alleviated by employing the ferroelectric capacitor in CMOS back-end-of-line (BEOL). In addition, the MFMIS configuration should provide the flexibility/opportunity to test various combinations between baseline device and ferroelectric capacitor, and thereby, helping to understand the hysteresis of the FeFET. Because of this configuration, it was observed that the hysteresis window of FE-FDSOI/FE-FinFET is decreased as (i) the top-electrode area of ferroelectric capacitor is increased, and/or (ii) the device dimensions of baseline FET are decreased.

## Data Availability

The datasets used and/or analysed during the current study are available from the corresponding author on reasonable request.

## Abbreviations

FDSOI: Fully depleted silicon-on-insulator
FinFET: Fin-shaped field-effect transistor
FE-FDSOI: Ferroelectric capacitor + FDSOI
FE-FinFET: Ferroelectric capacitor + FinFET
PCM: Phase-change memory
RRAM: Resistive change memory
CBRAM: Conductive bridge type memory
FeFET: Ferroelectric field-effect transistor
CMOS: Complementary metal–oxide–semiconductor
LSMO: La_0.7_Sr_0.3_MnO_3_
NSO: NdScO_3_
PZT: Pb(Zr_0.2_Ti_0.8_)O_3_
PLD: Pulsed laser deposition
BOX: Buried oxide
EOT: Equivalent oxide thickness
